# Quantitative computed tomography analysis of the airways in patients with cystic fibrosis using automated software: correlation with spirometry in the evaluation of severity*

**DOI:** 10.1590/0100-3984.2015.0145

**Published:** 2016

**Authors:** Marcel Koenigkam Santos, Danilo Lemos Cruvinel, Marcelo Bezerra de Menezes, Sara Reis Teixeira, Elcio de Oliveira Vianna, Jorge Elias Júnior, José Antonio Baddini Martinez

**Affiliations:** 1 PhD, MD, Radiologist, Collaborating Professor at the Center for Imaging Sciences and Medical Physics of the Hospital das Clínicas da Faculdade de Medicina de Ribeirão Preto da Universidade de São Paulo (HCFMRP-USP), Ribeirão Preto, SP, Brazil.; 2 MD, Radiology Specialist at the Center for Imaging Sciences and Medical Physics of the Hospital das Clínicas da Faculdade de Medicina de Ribeirão Preto da Universidade de São Paulo (HCFMRP-USP), Ribeirão Preto, SP, Brazil.; 3 PhD, MD, Attending Pulmonologist in the Pulmonology Sector of the Department of Clinical Medicine of the Faculdade de Medicina de Ribeirão Preto da Universidade de São Paulo (FMRP-USP), Ribeirão Preto, SP, Brazil.; 4 PhD, MD, Attending Radiologist at the Center for Imaging Sciences and Medical Physics of the Hospital das Clínicas da Faculdade de Medicina de Ribeirão Preto da Universidade de São Paulo (HCFMRP-USP), Ribeirão Preto, SP, Brazil.; 5 PhD, MD, Pulmonologist, Professor in the Pulmonology Sector of the Department of Clinical Medicine of the Faculdade de Medicina de Ribeirão Preto da Universidade de São Paulo (FMRP-USP), Ribeirão Preto, SP, Brazil.; 6 PhD, MD, Radiologist, Professor at the Center for Imaging Sciences and Medical Physics of the Hospital das Clínicas da Faculdade de Medicina de Ribeirão Preto da Universidade de São Paulo (HCFMRP-USP), Ribeirão Preto, SP, Brazil.

**Keywords:** Cystic fibrosis, Tomography, X-ray computed, Airway remodeling/physiology

## Abstract

**Objective:**

To perform a quantitative analysis of the airways using automated software, in computed tomography images of patients with cystic fibrosis, correlating the results with spirometric findings.

**Materials and Methods:**

Thirty-four patients with cystic fibrosis were studied-20 males and 14 females; mean age 18 ± 9 years-divided into two groups according to the spirometry findings: group I (*n* = 21), without severe airflow obstruction (forced expiratory volume in first second [FEV1] > 50% predicted), and group II (*n* = 13), with severe obstruction (FEV1 ≤ 50% predicted). The following tracheobronchial tree parameters were obtained automatically: bronchial diameter, area, thickness, and wall attenuation.

**Results:**

On average, 52 bronchi per patient were studied. The number of bronchi analyzed was higher in group II. The correlation with spirometry findings, especially between the relative wall thickness of third to eighth bronchial generation and predicted FEV1, was better in group I.

**Conclusion:**

Quantitative analysis of the airways by computed tomography can be useful for assessing disease severity in cystic fibrosis patients. In patients with severe airflow obstruction, the number of bronchi studied by the method is higher, indicating more bronchiectasis. In patients without severe obstruction, the relative bronchial wall thickness showed a good correlation with the predicted FEV1.

## Abstract (pt)

**Objetivo:**

Realizar a análise quantitativa das vias aéreas utilizando programa automático, em imagens de tomografia computadorizada de pacientes com fibrose cística, correlacionando com a espirometria.

**Materiais e Métodos:**

Foram estudados 34 pacientes com fibrose cística - 20 masculinos e 14 femininos; idade de 18 ± 9 anos -, divididos em dois grupos segundo a espirometria: grupo I (*n* = 21) - sem obstrução grave ao fluxo aéreo (volume expiratório forçado no primeiro segundo [VEF1] > 50% previsto); grupo II (*n* = 13) - com obstrução grave (VEF1 ≤ 50% previsto). Foram automaticamente obtidos: diâmetro, área, espessura e atenuação da parede da árvore traqueobrônquica.

**Resultados:**

Na média, foram estudados 52 brônquios por paciente. O número de brônquios analisados foi maior no grupo II. A correlação com a espirometria foi melhor no grupo I, principalmente entre a espessura relativa da parede da terceira a oitava geração brônquica e o VEF1 previsto.

**Conclusão:**

A análise quantitativa das vias aéreas em imagens de tomografia computadorizada pode ser útil na avaliação da gravidade da doença na fibrose cística. Nos pacientes com obstrução grave ao fluxo aéreo, o número de brônquios estudados pelo método é maior, indicando mais bronquiectasias. Nos pacientes sem obstrução grave, a espessura relativa da parede dos brônquios tem boa correlação com o VEF1 previsto.

## INTRODUCTION

Cystic fibrosis (CF) is a chronic autosomal recessive disease that affects the epithelial cells of multiple organs, especially the respiratory tract and exocrine pancreas, and is the leading cause of death from inherited disease in white populations. Multiple genetic mutations have been associated with this disease, which in the lungs result in deficient chloride secretion and increased absorption of sodium by airway epithelial cells. Patients with CF present with secretions of greater viscosity, with impaired mucociliary clearance, chronic obstruction, and persistent, recurrent bacterial infections, factors that lead to a bronchopulmonary disease with high morbidity and mortality. Chronic inflammation of the airways leads to deformity and dilation of the bronchi, together with thickening of the bronchial wall. The resulting bronchiectasis, in turn, predisposes to chronic bacterial infection, creating a vicious cycle of tissue damage^(1-3)^. Although there is still no cure, improvements in the treatment of patients with CF have led to a significant increase in life expectancy, which is now approximately 40 years in most developed countries^(4)^.

Imaging tests play a very important role not only in diagnosis but also in the clinical follow-up of CF-related bronchopulmonary disease and are used in assessing the severity of the disease, characterizing its regional distribution, and detecting complications (infectious and noninfectious), as well as for monitoring the response to treatment. Currently, high-resolution multidetector computed tomography (MDCT) is considered the best imaging method for the morphological characterization of bronchial and pulmonary changes, being able to evaluate distal airways and with better accuracy than that of plain radiography and magnetic resonance imaging^(5,6)^. MDCT has been even suggested as a valid method for the characterization of outcomes in clinical trials and therapeutic interventions^(7,8)^.

The main features of CF on CT scans are bronchial dilation and deformity (bronchiectasis), bronchial wall thickening, accumulation of secretion (mucoid impaction and small airway opacities), atelectasis, and changes in pulmonary attenuation secondary to bronchial obstruction and hypovolemia^(8)^. Although various visual grading systems (scores) for imaging tests have been described to monitor the disease more objectively, such systems have been little used in clinical practice, mainly due to the complexity of their application^(9)^. Pulmonary function tests, especially spirometry, are used in the clinical follow-up of patients with CF. Regardless of the disease pattern (obstructive or restrictive), forced expiratory volume in first second (FEV1) is considered the most important parameter, representing a prognostic marker and predictor of mortality^(10)^. However, we know that pulmonary function tests are not sensitive to early changes in the airways and lung parenchyma, as well as being unable to show regional and compartmentalized distribution of the disease, characteristics that can have implications in targeted therapies^(11)^.

In view of the therapeutic improvement and survival gain of patients with CF, resulting in a prolonged period of serial follow-up, it is necessary to find parameters that are reliable and reproducible; that is, quantitative and objective for proper assessment of disease severity and treatment response. Recent studies have demonstrated that quantitative analysis of the airways on MDCT images using dedicated software is able to identify CF-related morphological changes that correlate well with functional parameters^(12,13)^.

The aim of this study was to quantitatively assess morphological changes in airways on MDCT scans, using a computer program with fully automated analysis capabilities in a population of patients with CF being followed clinically at our hospital, correlating the results with those of spirometry.

## MATERIALS AND METHODS

### Patients

This study was approved by the local research ethics committee. Because this was a retrospective study based on tests already carried out in patients in whom those tests were clinically indicated for the monitoring/assessment of CF, a waiver of informed consent was granted.

We retrospectively analyzed the electronic medical records and MDCT scans of patients (children and adults) with CF who were followed clinically at our referral hospital. Spirometry and CT were ordered as part of the routine clinical evaluation of these patients, so that there was no record of the investigation of acute exacerbation, infection, or suspicion of other acute complications. We analyzed the records of patients with symptoms and signs typical of CF, a family history of the disease, and diagnostic confirmation (mainly by determination of sweat chloride levels). We included consecutive patients whose spirometry and MDCT scans had been performed at our facility, in the same devices, within a four-week period. We excluded cases in which the spirometry or CT examinations were of poor technical quality, considered inadequate for diagnosis and analysis. Most spirometry tests were performed with a KoKo spirometer (PDS Instrumentation, Inc., Louisville, CO, USA), although a Vitatrace VT 130 spirometer (Pró Médico Ltda., Rio de Janeiro, Brazil) was employed in six cases (all children). The performance of the tests and characteristics of the apparatus were in accordance with the American Thoracic Society standards for spirometry.

Patients were divided into two groups according to the presence or absence of severe airflow obstruction^(14)^, based on the measurement of FEV1 as a percentage of the predicted value (FEV1%) obtained in spirometry after bronchodilator use: group I-patients without severe airflow obstruction (FEV1% > 50); and group II-patients with severe airflow obstruction (FEV1% ≤ 50).

### Chest CT

All CT examinations were performed in a 16-detector MDCT scanner (Brilliance Big Bore; Philips, Amsterdam, the Netherlands) and followed the same protocol. Patients underwent high-resolution CT without intravenous administration of iodinated contrast, scanning across the chest in the caudocranial direction, with volumetric acquisition of 1-mm slices at full inspiration. Other typical parameters of acquisition were a tube voltage of 120 kVp; a reference tube current of 110-130 mAs; a gantry rotation time of 0.3-0.7 s; and an ideal exposure to radiation of < 5 mSv. Volumetric acquisitions were reconstructed with a soft/standard filter and with a hard filter, with 1-mm slices and a 1-mm reconstruction interval.

### Obtaining airway measurements

Quantitative analysis of the airways on CT images was performed using the Yacta program, version 2.0^(15)^, installed on a computer in the image processing lab of our department and connected to the network servers of the hospital. Therefore, the images are sent directly from the scanner to be analyzed by the program.

**Figure 1:**
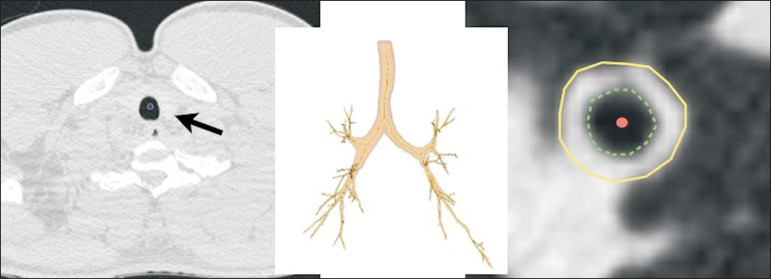
For obtaining airway measurements with the automated program, the first step is identification of the tracheal lumen (arrow), followed by segmentation (anatomical separation) of the tracheobronchial tree. A center line is calculated within the airway to identify the true axial plane of the bronchus, then the inner borders (dotted line) and outer borders (continuous line) are identified.

The Yacta program works automatically, not requiring the intervention of a user at any stage of the process. Image analysis takes 4-9 minutes after submission for processing. Initially, Yacta segments (i.e., anatomically separates) the airways, the right lung, the left lung, and the lung lobes, based on a computerized algorithm for the recognition of structures, densities, and anatomical thresholds. A center line is calculated, within the airway, from the trachea to the distal bronchi and is used as a reference for measurements in the true transverse plane of the bronchus (perpendicular to the line axis). After the transverse plane of the bronchus has been identified, different algorithms can be used for recognition of inner and outer borders, as well as to calculate the caliber and wall thickness of the bronchus, the most widely used being the full-width-at-half-maximum determination and the integral-based method. In this study we used the integral-based method (Figure 1), which has been shown to be more reliable for anatomical measurements^(13,15)^.

The following measurements of the airways were obtained: the number of bronchi analyzed; the overall airway diameter (distance between the outer borders of the bronchus, in mm); area of the lumen (area between the inner borders, in mm^2^); wall thickness (distance between the inner and outer edges, in mm); relative wall thickness (RWT) of the bronchus (ratio of wall thickness to overall diameter, in %); RWT of the third to eighth bronchial generation (RWT3-8); and maximum attenuation of the bronchial wall (maximum density points between the inner and outer edge, in Hounsfield units). All of these measures are obtained for *n* bronchi of the segmented tracheobronchial tree, and the program calculates an average for the entire tree, although it also identifies the values according to the order of bronchial generation.

### Statistical analysis

All data were organized and analyzed on a personal computer with a spreadsheet program (Microsoft Excel 2011) and a statistical analysis program (GraphPad Prism 5.0). The Shapiro-Wilk test was used in order to verify the normal distribution of the variables. We used unpaired t-tests to compare the data between groups I and II, at a significance level of 95% (*p* < 0.05). Correlations between airway measures and spirometric parameters were assessed with Pearson's correlation coefficient.

## RESULTS

We evaluated 34 patients with CF (14 females and 20 males), with a mean age of 18 ± 9 years (range, 7-43 years). Group I comprised 21 patients, and group II comprised 13 patients. The mean age of the patients was significantly lower in group I than in group II (14.3 ± 6 *versus* 23.9 ± 10.5 years). Table 1 summarizes the spirometry results. Groups I and II showed significant differences in pulmonary function measures, including FEV1, FEV1%, forced vital capacity (FVC, in liters) and as a percentage of the predicted value), and the Tiffeneau index (FEV1/FVC ratio). Only FVC values did not differ significantly between groups I and II (*p* = 0.25).

**Table 1:** Spirometry results of CF patients.

	Tiffeneau index	FEV1 (L)	FEV1%	FVC (L)	FVC%
All patients	0.71 ± 0.15	2.0 ± 1.8	71.3 ± 30.2	2.8 ± 1.0	81.5 ± 24.3
Group I	0.81 ± 0.9	2.3 ± 0.8	90.6 ± 20.8	2.9 ± 1.0*	95.7 ± 17.9
Group II	0.55 ± 0.7	1.4 ± 0.3	40.1 ± 8.9	2.5 ± 0.9*	58.5 ± 12.8

FEV1, forced expiratory volume in first second; FEV1%, FEV1 in percentage of predicted; FVC, forced vital capacity; FVC%, FVC in percentage of predicted; Tiffeneau index, FEV1/FVC.

* Only the values of FVC did not differ significantly different between groups I and II (*p* = 0.25).

**Figure 2:**
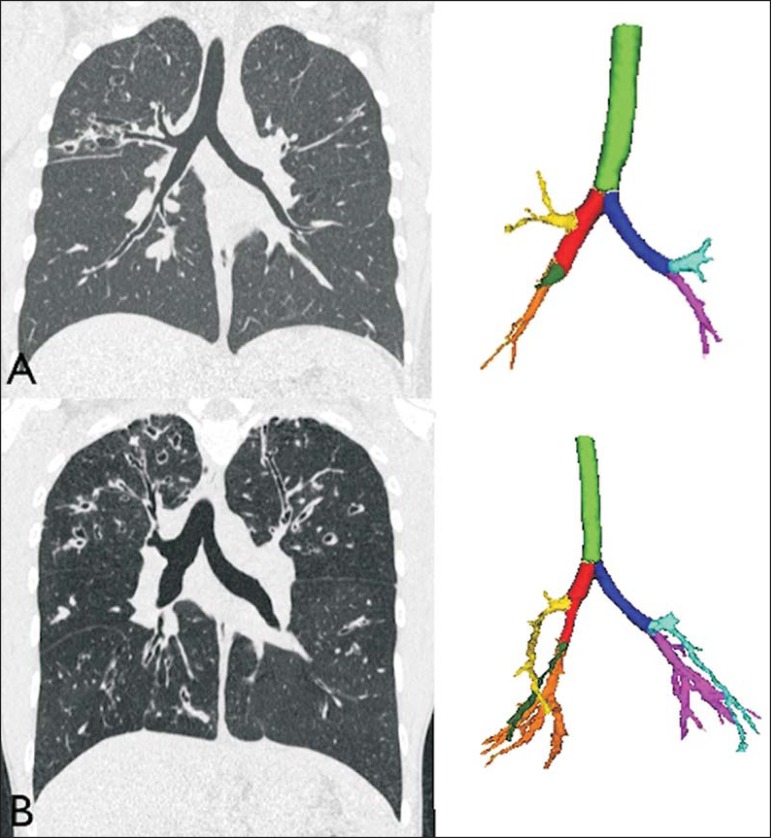
Examples of two CF patients, one from group I (**A**, FEV1% = 69.6) and another from group II (**B**, FEV1% = 34.5). Coronal CT scans with a lung window show greater bronchiectasis in the group II patient. The three-dimensional figures representing the automated bronchial segmentation illustrate that the number of bronchi found and analyzed was greater in the group II patient than in the group I patient (91 vs. 49).

The Yacta program was able to segment and analyze the scans of all 34 patients included in the study, obtaining quantitative airway measures. On average, we studied 52 bronchi per patient. Considering all patients, we found that the mean overall diameter of the bronchi was 7.8 mm, the mean bronchial wall thickness was 2.2 mm, and the RWT was 83% (RWT3-8 of 85%). Table 2 summarizes the CT-acquired airway measurements after automated analysis. When comparing groups I and II, we found that the only variable showing a statistical difference between groups was the number of bronchi evaluated (Figure 2), which was higher in group II (*p* < 0.01).

**Table 2:** Results of the quantitative analysis of airways on MDCT images of CF patients.

	Number of bronchi studied	Total diameter of the bronchus (mm)	Area of the lumen (mm^2^)	Mean wall thickness (mm)	Relative wall thickness (%)	RWT3-8 (%)	Maximum wall attenuation (UH)
All patients	52 ± 22	7.8 ± 0.9	8.8 ± 3.9	2.2 ± 0.2	83.3 ± 1.9	85.1 ± 2.0	-375 ± 121
Group I	44 ± 17*	7.6 ± 0.7	8.7 ± 3.6	2.2 ± 0.1	83.4 ± 2.3	85.0 ± 2.0	-398 ± 138
Group II	64 ± 24*	8.0 ± 1.2	8.9 ± 4.5	2.3 ± 0.1	83.0 ± 1.2	85.2 ± 1.4	-337 ± 79

RWT3-8, relative wall thickness of the third to eighth bronchial generation.

* The only variable that showed statistically significant differences between groups I and II was the number of bronchi studied (*p* < 0.01).

**Figure 3:**
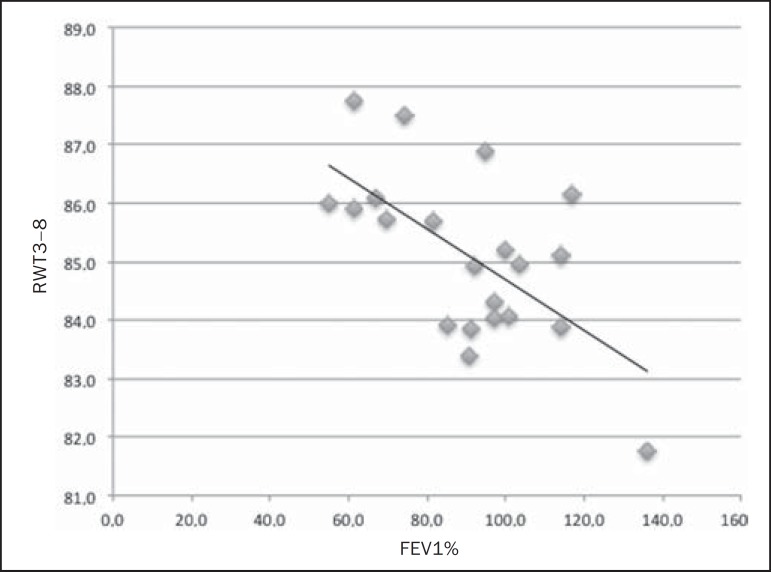
Graphic illustrating the correlation between the RWT3-8 measured by CT and the FEV1% in group I patients (*R* = -0.62; *p* < 0.01).

Although other quantitative airway measures did not differ significantly between the groups, there were significant correlations between these measures and the spirometry results. In group I, we identified that correlation between RWT3-8 value and FEV1% was significant (*R* = -0.62; *p* < 0.01), greater RWT3-8 translating to lower FEV1% (Figure 3). In group II, we identified no significant correlation coefficient. Observing correlation coefficient values between the groups, we noted a trend toward better correlation of measures in group I. Table 3 shows the Pearson's correlation coefficients for the two groups with respect to FEV1%.

**Table 3:** Correlation coefficients for comparisons between MDCT-acquired quantitative measurements of the airways and FEV1%.

	Correlation coefficient	
Measure	Group I	Group II
Number of bronchi	-0.35	0.12
Overall diameter	-0.03	0.05
Area of the lumen	-0.06	-0.16
Wall thickness	-0.25	0.16
Relative wall thickness	-0.27	0.22
Relative wall thickness of the third to eight generation	-0.62*	0.29
Maximum wall attenuation	-0.23	0.21

* The correlation between RWT3-8 and FEV1% was stronger in group I than in group II, and the difference between the two groups was statistically significant (*p* < 0.01).

## DISCUSSION

We studied a sample of 34 patients diagnosed with CF who had undergone MDCT and spirometry at our hospital, using a fully automated quantitative analysis program to measure airways in high-resolution CT images of the chest. The program was able to analyze a large number of bronchi in all of the patients studied and to identify changes related to CF. In patients with severe airflow obstruction, a greater number of bronchi were evaluated, reflecting greater bronchiectasis in these patients. In patients without severe airflow obstruction, we found a good correlation between RWT3-8 and FEV1%.

The technological evolution of CT equipment, with the development of the multidetector technique, has made it possible to achieve high-resolution volumetric acquisition of images of the entire chest during a single breath-hold. The current image quality allows detailed assessment of the morphology of the lung parenchyma and airways down to the subsegmental level^(16)^. In the assessment of bronchopulmonary diseases, many studies have demonstrated that CT is superior to other diagnostic tools in that it is able to identify changes earlier, being more sensitive in detecting subtle changes, and allows the regional/compartmental assessment of the disease^(17)^. In CF, for example, it has been demonstrated that CT is able to detect progression of bronchopulmonary changes in patients without significant changes in the parameters of pulmonary function tests^(18)^.

Compared with the traditional qualitative analysis of imaging tests, quantitative evaluation is more objective, less dependent on the reader/evaluator, and has better reproducibility. Primarily in the study of chronic obstructive pulmonary disease and asthma, the quantitative analysis of CT scans, done automatically or semi-automatically, has shown good accuracy and good correlation with functional tests^(19-22)^. In attempts to standardize imaging evaluation of CF, various visual grading systems (scores) have been devised for use in plain radiography, CT, and MRI. However, those systems have been little used in clinical practice, mainly due to the complexity of their application and the differences among them^(23)^. Recent studies have demonstrated that quantitative analysis of the airways is also able to detect changes in CF^(12,13)^.

In the present study, we evaluated MDCT images obtained for 34 patients with CF. The program used was able to segment and perform a fully automated analysis of the airways in all cases, evaluating an average of 52 bronchi per patient. Although the first studies of quantitative analyses of the airways described the measurements in one or only a few bronchi, performed manually or semiautomatically^(24)^, automated evaluation of a large number of bronchi, as performed in the present study, allows a more accurate and reproducible analysis of airway disease, considering the lobar and segmental distribution of the disease and the different bronchial generations.

The caliber and wall thickness of bronchi diminish progressively along the tracheobronchial tree, from the main bronchi toward the peripheral bronchioles; that is, the higher the order of bronchial generation is, the smaller is the bronchial caliber and the greater is the thickness of the airway. The normal bronchial wall thickness is 0.2-0.3 mm for subsegmental bronchi and 1.0-1.5 mm for lobar/segmental bronchi. The normal bronchial caliber is 5-8 mm for segmental bronchi, whereas it is = 1 mm for subsegmental bronchi and bronchioles^(13,25)^. The size and thickness of the bronchial walls are related to airflow limitation in obstructive diseases^(19)^. For example, a bronchial wall thickness ³ 1.75 mm in segmental bronchi is associated with an increased risk of acute exacerbations in patients with chronic obstructive pulmonary disease^(26)^. Among normal individuals, the mean RWT of the bronchial wall is < 50%, varying with age and the order of bronchial generation studied^(13,27)^. In the present study, the program employed in the automated analysis of the airways was able to identify and objectively measure the CF-related dilation and thickening of the bronchial walls. Among the 34 CF patients evaluated here, the mean bronchial diameter was 7.8 mm, the mean bronchial wall thickness was 2.2 mm, and the mean RWT was 83%.

In our comparison between patients with and without severe airflow obstruction, the only quantitative airway variable that showed a significant difference was the number of bronchi analyzed. Wielpütz et al.^(13)^ reported that the number of bronchi studied was greater in CF patients than in controls. The loss of physiological tapering of the airways is related to the development of bronchiectasis and allows the automated program to advance further in the segmentation by identifying and measuring a greater number of bronchi. The relationship of this variable (greater number of automatically identified bronchi) with the presence of severe airflow obstruction in patients with CF has not been previously described in the literature.

Although we found no significant differences between the two groups of CF patients in terms of the various airway measurements, those measurements correlated well with the spirometric parameters. The main finding was that we identified good correlation between RWT3-8 and FEV1% in the patients without severe airflow obstruction (*R* = -0.62), that correlation being statistically significant and stronger than the correlation identified in the patients with severe airflow obstruction. Although there is as yet no consensus in the medical literature as to the most appropriate airway measures to be studied on CT images^(28)^, our study shows that determining the RWT of the bronchi can be more advantageous than is the determination of other measures. The linear measures of bronchial caliber, area of the bronchial lumen, and bronchial thickness vary according to the order of bronchial generation and anthropometric parameters (sex, age, height, and weight), whereas RWT is a proportional measure and probably tends to be more constant in this population, regardless of other constitutional variables. Discarding the first and second bronchial generations (main and lobar bronchi), we focused our analysis on the small airways (subsegmental bronchi), where the initial and most important changes in obstructive pulmonary disease occur^(29)^. The weaker correlation between tomography measurements and spirometric parameters in the group of patients with severe airflow obstruction suggests that the presence of changes other than bronchiectasis, such as mucoid impaction, atelectasis, and parenchymal destruction, contributes to the functional impairment associated with the disease. These changes are not evaluated by the program for the automated analysis of the airways on CT images.

This study has limitations. The spirometry tests were not all performed in the same device and by the same technician, which would be the ideal situation in order to avoid any bias related to the performance of the test. We did not use spirometric monitoring of inspiration during the performance of CT scans, which could confirm the acquisition of images at full inspiration, especially in younger patients. The degree of inspiration can affect quantitative measures of the lungs and airways on CT images. Despite the advanced algorithm implemented by the automated program, the analysis of bronchi of the distal segments also creates a relative selection bias because it preferably detects the airways that are dilated. Other limitations of computational analysis of the airways are inherent to CT imaging, because small differences in attenuation values and discrete changes in the lumen can complicate correct the identification of the bronchial borders and the differentiation between wall thickening and partial obstruction by mucus or adjacent structures. Morphological analysis of distal bronchi to the ninth bronchial generation is not possible with the MDCT techniques currently available^(30)^. It should also be borne in mind that the generations of segmented airways based on automated bifurcations are not exactly equal to anatomical generations. As for the functional correlation, we did not analyze values of forced expiratory flow, a measure that can show initial obstructive change of the airway. For some patients, we did not have access to plethysmography-acquired lung volumes, which could also be correlated with the parameters obtained by CT. Finally, we did not adjust airway measures for patient height and weight, which, especially in comparisons involving adults and children, could increase the accuracy of the analysis.

## CONCLUSION

Quantitative analysis of the airways on CT images can be useful in disease severity assessment in CF. In patients with severe airflow obstruction, the method allows a greater number of bronchi to be studied, which makes it more sensitive in detecting bronchiectasis. In patients without severe airflow obstruction, the RWT3-8 correlated well with FEV1%, which is the spirometric parameter most widely used in clinical follow-up and considered to have prognostic value for CF.
