# Erratum

**DOI:** 10.1590/0100-3984.2015.0145er

**Published:** 2016

In the article "Quantitative computed tomography analysis of the airways in patients with cystic fibrosis using automated software: correlation with spirometry in the evaluation of severity", DOI No.: http://dx.doi.org/10.1590/0100-3984.2015.0145, published in Radiol Bras. Nov/Dez 2016;49(6):351- 357, in the page 351, right column:

where is read:

Received July 30, 2014. Accepted after revision November 4, 2015.

it should be written:

Received July 30, 2015. Accepted after revision November 4, 2015.

